# Supplementary material for: Giant thermal expansion and α-precipitation pathways in Ti-alloys
Source: Nat Commun. 2017 Nov 10;8:1429. doi: 10.1038/s41467-017-01578-1 (PMC5681671; doi:10.1038/s41467-017-01578-1)
Supplement: Supplementary file 1 — Supplementary Information [file 41467_2017_1578_MOESM1_ESM.pdf]

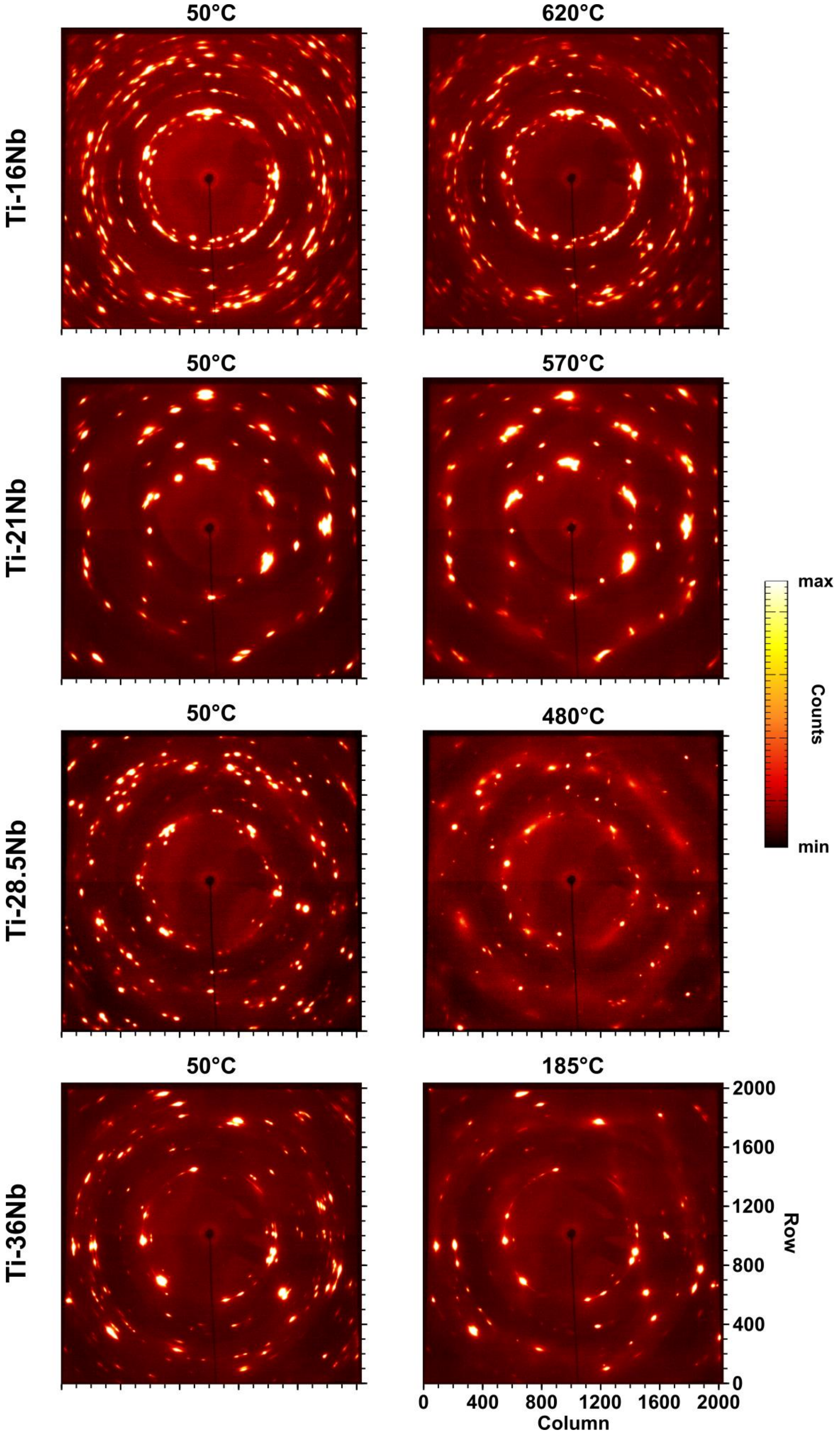


**Supplementary Figure 1: Raw untreated 2-dimensional diffraction patterns showing the entire detector area.** For each alloy the image in the left column corresponds to the initial state at 50°C. The images in the right column correspond to decomposition of α'' martensite for Ti-16Nb and Ti-21Nb, to $\alpha''_{iso}$ formation for Ti-28.5Nb and to reversion of α'' martensite for Ti-36Nb.
